# Supplementary material for: Understanding pectin cross-linking in plant cell walls
Source: Commun Biol. 2025 Jan 17;8:72. doi: 10.1038/s42003-025-07495-0 (PMC11748717; doi:10.1038/s42003-025-07495-0)
Supplement: Supplementary file 1 — Supplementary Information [file 42003_2025_7495_MOESM1_ESM.pdf]

Understanding Pectin Cross-linking in Plant Cell Walls SUPPLEMENTARY INFORMATION

Irabonosi Obomighie[1], Iain J. Prentice[2], Peter Lewin-Jones[3], Fabienne Bachtiger[2], Nathan Ramsay[1], Chieko Kishi-Itakura[1], Martin W. Goldberg[1], Tim J. Hawkins[1], James Sprittles[3], Heather Knight[1*] and Gabriele C. Sosso[2*]

[1]Department of Biosciences and Durham Centre for Crop Improvement Technology, University of Durham, Durham, DH1 3LE, United Kingdom.
[2]Department of Chemistry, University of Warwick, Coventry, CV4 7AL, United Kingdom.
[3]Warwick Mathematics Institute, University of Warwick, Coventry, CV4 7AL, United Kingdom.

*Corresponding author(s). E-mail(s): p.h.knight@durham.ac.uk; g.sosso@warwick.ac.uk;

Computational details

Reproducibility of the cross-linking molecular dynamics simulations

We report in Supplementary Table 1 some information re: the reproducibility of our results in terms of the structural and dynamical properties of the HG aggregates we have observed by means of molecular dynamics simulations. Specifically, the mean and standard deviation values reported in Supplementary Table 1 have been obtained from 15, statistically independent molecular dynamics simulations (each 60 ns long) of the cross-linking of (8) HG chains of composition DDPDPPDP (see main text).

Supplementary Table 1 Reproducibility of the cross-linking molecular dynamics simulations. We report the mean and standard deviation re: the average number of either Ca^{2+} links (CL) or hydrogen bond links (HB) obtained from 15, statistically independent molecular dynamics simulations (each 60 ns long) of the cross-linking of (8) HG chains of composition DDPDPPDP (see main text). We also report the same metrics re: the average lifetime of CL- or HB-linked HG aggregates as wells as the average size of the biggest HG within a given simulation.

	mean	standard deviation
ave. CLs	9.54	0.83
ave. HBs	1.79	1.05
ave. CL lifetime / ns	7.16	1.56
ave. HB lifetime / ns	1.66	0.39
ave. size of biggest aggregate	7.71	0.17

The impact of the usage of different water models

Initially, we have performed molecular dynamics (MD) simulations of HG in water utilising the CHARMM36 force field (HG chains) in combination with the TIP4P/Ice water model (water molecules). This is because previous work by us as well as other authors [1–3] has demonstrated the validity of this particular combination of force fields in describing the interactions of bio molecules with water, especially when working with supercooled liquid water (which we intend to explore in the context of HG in future work).

However, at ambient temperature and pressure, the (self-)diffusion coefficient of TIP4P/Ice water is 1.2 nm^2/ns [4], which is substantially lower than the experimental value of 2.3 nm^2/ns [5]. In contrast, the diffusion coefficient of (the original) TIP4P water model at the same conditions of temperature and pressure is 2.4 nm^2/ns [6], which is much closer to the experimental value.

Whilst we found that the slower water dynamics of the TIP4P/Ice water model compared to that of the TIP4P water model does not have any impact on the results reported in this work, using the TIP4P/Ice water model is much more computationally expensive than using the TIP4P water model,

particularly when studying the aggregation of long HG chains in water. This is because the slow diffusion of TIP4P/Ice water results in a slower diffusion of the HG chains as well, which in turns leads to much longer simulations times to observe the aggregation process.

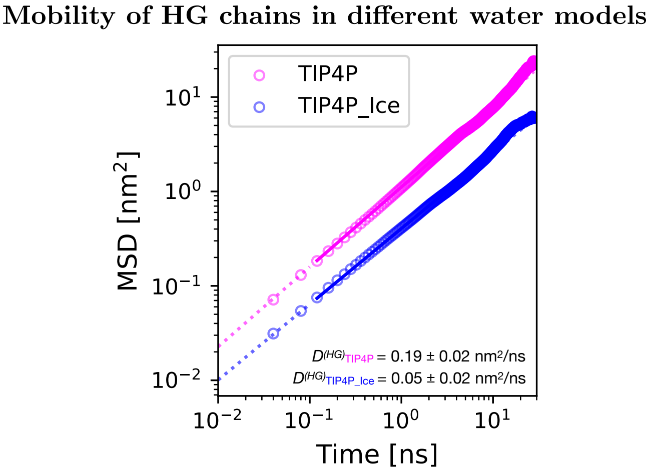

Fig. S1 *Mean squared displacement (MSD, see text) for the HG chains in either TIP4P or TIP4P/Ice water. These results have been computed for systems containing 8 40-unit HG chains solvated in ∼200,000 water molecules, over 30 ns long molecular dynamics trajectories.*

For the purposes of offering a quantitative comparison, we report in Fig. S1 the mean square displacement (MSD) of (the centre of mass of) the HG chains, computed for the same HG/water system (8 HG chains, 40-unit each - same system depicted in Fig. 3 in the main text) at ambient temperature and pressure, as a function of time - using either the TIP4P or the TIP4P/Ice water model. The resulting diffusion coefficients, 0.19 and 0.05 nm^2/ns for HG in TIP4P and TIP4P/Ice water, respectively, demonstrate the impact of the slow dynamics of the TIP4P/Ice water at room temperature.

The MSD has been computed by means of the Einstein formula [7]:

$$MSD = \left\langle \frac{1}{N} \sum_{i=1}^{N} \mid \mathbf{r}_i^{COM}(t) - \mathbf{r}_i^{COM}(t_0) \mid^2 \right\rangle_{t_0} , \quad (1)$$

where N is the number of chains in the system, $\mathbf{r}_i^{COM}(t)$ is the position of the center of mass of the i-th chain at time t and $\langle \dots \rangle_{t_0}$ refers to an average over different time origins t_0 which we have selected every 0.5 ns to ensure uncorrelated statistics.

From the MSD, one can straightforwardly derive the diffusion coefficient D as [7]:

$$D = \frac{1}{2d} \lim_{t\to\infty} \frac{d}{dt} MSD, \tag{2}$$

where d is the dimensionality of the system (in our case, 3). For practical purposes, one fits the linear regime of the MSD (in our case, from 0.1 to 10 ns), which can be better appreciated via a log-log plot such as the one reported in Fig. S1. Note that the value of D does depend on the size of the simulation box [8], but this is largely irrelevant for the sake of a relative comparison such as the one we interested in here. The uncertainty (± 0.02 nm^2/ns) associated with the estimate of the diffusion coefficients has been derived by starting from the uncertainty related to the (linear) fitting of the MSD.

Thus, for the purposes of computational efficiency (which in turned allowed us to accumulate a robust statistics in terms of the results presented in the main paper), we have chosen to adopt the TIP4P water model. We remark that this choice has no significant impact on the results presented in the main paper - in fact, we have explicitly verified this for the cross-linking results reported in Table 1 in the main paper (in the case of the PPPPPPPP and DDDDDDDD functionalisations, specifically).

Molecular dynamics simulations setup

We report in Supplementary Table 2 the details of the computational setup we have utilized to perform the molecular dynamics simulations of HG crosslinking reported in the main text. Each simulation is 40 ns long.

Supplementary Table 2 Computational setup with respect to the molecular dynamics simulations of HG crosslinking reported in the main text.

Pectin composition	N. of simulations	Box dimensions (nm)	N. of atoms	N. of water molecules	Salt concentration (mol/L)
PPPPPPPP	1	5.47	20580	4795	0.97
DDDDDDDD	1	5.53	21188	4979	0.31
MMMMMMMM	1	5.64	22532	5235	0.89
DDPDPPDP	1	5.54	21380	5023	0.16
DDMDMMDM	1	5.66	22800	5354	0.15
DPDPDPDP	1	5.49	20836	4886	0.16
DMDMDMDM	1	5.66	22744	5340	0.15
DDPPDDPP	1	5.49	20972	4921	0.16
DDMMDDMM	1	5.67	22720	1200	0.15
PPDDDDPP	1	5.55	21492	1300	0.16
MMDDDDMM	1	5.59	22448	1400	0.15
DDPPPPDD	1	5.56	21956	1500	0.15
DDMMMMDD	1	5.50	20888	1600	0.16
DPPDDPPD	1	5.55	21440	1700	0.16
DMMDDMMD	1	5.48	20852	1800	0.16
PDDPPDDP	1	5.48	20832	1900	0.16
MDDMMDDM	1	5.61	22692	2000	0.15
DDDDPPPP	1	5.54	21444	2100	0.16
DDDDMMMM	1	5.66	22728	2200	0.15
dddd	1	19.05	918716	228113	0.04
mmmm	1	19.24	944220	234209	N/A
dddp	1	19.05	918844	228135	0.03
dddm	1	19.05	918812	228067	0.03
ddpd	1	19.06	919016	228178	0.03
ddmd	1	19.05	918852	228077	0.03
ddpp	1	19.06	918984	228160	0.02
ddmm	1	19.06	918828	228001	0.02
dppd	1	19.06	919216	228218	0.02
dmmd	1	19.06	918840	228004	0.02
pddp	1	19.06	919064	228180	0.02
mddm	1	19.23	943956	234283	0.02
dpdp	1	19.06	919028	228171	0.02
dmdm	1	19.06	918824	228000	0.02
dppp	1	19.06	919296	228228	0.01
dmmm	1	19.06	919108	228001	0.01
pdpp	1	19.06	919312	228232	0.01
mdmm	1	19.23	943868	234191	0.01

Metadynamics simulations setup

We report in Supplementary Table 3 the details of the computational setup we have utilized to perform the metadynamics simulations reported in the main text.

Supplementary Table 3 Computational setup with respect to the metadynamics simulations. In terms of the composition of our HG chains, we have protonated -COOH groups (**P**), de-protonated -COO$^-$ groups (**D**) and methylated carboxyl groups (**M**).

System	N. of simulations	Box dimensions (nm)	N. of atoms	N. of water molecules	Salt concentration (mol/L)	Pectin composition
Ca^{2+} - H_2O	1	5.38	21257	5233	0.01	2xPPPPPPPD
Ca^{2+} - COO^-	1	5.38	21257	5233	0.01	2xPPPPPPPD
CH_3-O	1	5.39	21288	5239	-	2xPPPPPPPM
COOH - COOH	1	5.54	23184	5704	-	2xMMMMMMMD
CN(Ca^{2+}-(4)COO^-)/CN(Ca^{2+}-H_2O)	1	5.32	20449	5031	0.0106	2xPPPPPPPD

References

[1] Lee, H.: Interactions of antifreeze proteins in TIP4P / Ice water and their dependence on force fields. PLOS one **07**, 1–17 (2018). https://doi.org/10.1371/journal.pone.0198887

[2] Mochizuki, K., Molinero, V.: Antifreeze Glycoproteins Bind Reversibly to Ice via Hydrophobic Groups. Journal of the American Chemical Society **140**(14), 4803–4811 (2018). https://doi.org/10.1021/jacs.7b13630

[3] Sosso, C.G., Whale, T.F., Holden, A.M., Pedevilla, P., Murray, B.J., Michaelides, A.: Unravelling the Origins of Ice Nucleation on Organic Crystals. Chemical Science **9**, 8077–8088 (2018). https://doi.org/10.1039/C8SC02753F

[4] Baran, , Rżysko, W., MacDowell, L.G.: Self-diffusion and shear viscosity for the TIP4P/Ice water model. The Journal of Chemical Physics **158**(6), 064503 (2023). https://doi.org/10.1063/5.0134932. Accessed 2024-08-07

[5] Krynicki, K., D. Green, C., W. Sawyer, D.: Pressure and temperature dependence of self-diffusion in water. Faraday Discussions of the Chemical Society **66**(0), 199–208 (1978). https://doi.org/10.1039/DC9786600199. Publisher: Royal Society of Chemistry. Accessed 2024-08-07

[6] Zlenko, D.V.: Computing the self-diffusion coefficient for TIP4P water. BIOPHYSICS **57**(2), 127–132 (2012). https://doi.org/10.1134/S0006350912020273. Accessed 2024-08-07

[7] Maginn, E.J., Messerly, R.A., Carlson, D.J., Roe, D.R., Elliot, J.R.: Best Practices for Computing Transport Properties 1. Self-Diffusivity and Viscosity from Equilibrium Molecular Dynamics [Article v1.0]. Living Journal of Computational Molecular Science **1**(1), 6324–6324 (2019). https://doi.org/10.33011/livecoms.1.1.6324. Number: 1. Accessed 2024-08-07

[8] Yeh, I.-C., Hummer, G.: System-Size Dependence of Diffusion Coefficients and Viscosities from Molecular Dynamics Simulations with Periodic Boundary Conditions. J. Phys. Chem. B **108**(40), 15873–15879 (2004). https://doi.org/10.1021/jp0477147. Publisher: American Chemical Society. Accessed 2024-08-07
